# Measuring Brain Temperature in Youth Bipolar Disorder Using a Novel Magnetic Resonance Imaging Approach: A Proof-of-concept Study

**DOI:** 10.2174/1570159X21666230322090754

**Published:** 2023-05-12

**Authors:** Yi Zou, Chinthaka Heyn, Anahit Grigorian, Fred Tam, Ana Cristina Andreazza, Simon J. Graham, Bradley J. Maclntosh, Benjamin I. Goldstein

**Affiliations:** 1 Department of Pharmacology, University of Toronto, Toronto, ON, Canada;; 2 Centre for Youth Bipolar Disorder, Centre for Addiction and Mental Health, Toronto, ON, Canada;; 3 Department of Medical Imaging, Sunnybrook Health Sciences Centre, Toronto, ON, Canada;; 4 Physical Sciences Platform, Sunnybrook Research Institute, Toronto, Canada;; 5 Department of Psychiatry, University of Toronto, Toronto, M5T 1R8, ON, Canada;; 6 Department of Medical Biophysics, University of Toronto, Toronto, ON, Canada;; 7 Heart and Stroke Foundation, Canadian Partnership for Stroke Recovery, Sunnybrook Research Institute, Toronto, ON, Canada;; 8 Hurvitz Brain Sciences Program, Sunnybrook Research Institute, Toronto, ON, Canada

**Keywords:** Youth, bipolar disorder, brain temperature, MRS, cerebral blood flow, mood

## Abstract

**Background:**

There is evidence of alterations in mitochondrial energy metabolism and cerebral blood flow (CBF) in adults and youth with bipolar disorder (BD). Brain thermoregulation is based on the balance of heat-producing metabolism and heat-dissipating mechanisms, including CBF.

**Objective:**

To examine brain temperature, and its relation to CBF, in relation to BD and mood symptom severity in youth.

**Methods:**

This study included 25 youth participants (age 17.4 ± 1.7 years; 13 BD, 12 control group (CG)). Magnetic resonance spectroscopy data were acquired to obtain brain temperature in the left anterior cingulate cortex (ACC) and the left precuneus. Regional estimates of CBF were provided by arterial spin labeling imaging. Analyses used general linear regression models, covarying for age, sex, and psychiatric medications.

**Results:**

Brain temperature was significantly higher in BD compared to CG in the precuneus. A higher ratio of brain temperature to CBF was significantly associated with greater depression symptom severity in both the ACC and precuneus within BD. Analyses examining the relationship of brain temperature or CBF with depression severity score did not reveal any significant finding in the ACC or the precuneus.

**Conclusion:**

The current study provides preliminary evidence of increased brain temperature in youth with BD, in whom reduced thermoregulatory capacity is putatively associated with depression symptom severity. Evaluation of brain temperature and CBF in conjunction may provide valuable insight beyond what can be gleaned by either metric alone. Larger prospective studies are warranted to further evaluate brain temperature and its association with CBF concerning BD.

## 1 INTRODUCTION

Bipolar disorder (BD) is a severe psychiatric disorder with typical onset in adolescence or young adulthood [1]. The disorder is multidimensional, including several subtypes with different neurobiological underpinnings and comorbidities [2-4]. Although the etiopathology of BD has not been fully elucidated and is likely multifactorial, mounting evidencepoints to a central role of mitochondrial dysfunction [5, 6] and anomalous energy metabolism [7] in the pathogenesis of BD. Specifically, changes to mitochondrial morphology [8], which plays an essential role in maintaining proper respiratory and metabolic function [9, 10], and the expression of genes regulating energy metabolism [11] have been frequently reported in adults with BD compared to healthy controls.

A recent review summarizing post-mortem tissue proteomic studies revealed alterations in the expression of proteins essential in the mitochondrial energy metabolism pathway in BD [7]. In addition, post-mortem studies have shown altered brain pH levels [12, 13] and reduced phosphocreatine/adenosine triphosphate ratio in this population [14], pointing to anomalous oxidative phosphorylation processes. A recent meta-analysis found that brain lactate levels were significantly elevated in BD patients compared to healthy controls [15], suggesting an alteration in mitochondria-mediated energy metabolism in the brain of BD patients. Finally, a post-mortem brain study revealed abnormal activity of complex I of the electron transport chain in adults with BD, providing direct evidence of altered brain energy production [16].

Importantly, studies utilizing *in vivo* magnetic resonance spectroscopy (MRS) have found altered energy metabolism in BD and alterations in relation to symptom severity in BD in both adults and youth. In adults, there is evidence that, compared to healthy controls, brain pH is lower during euthymia and higher during depression or mania [17, 18]. In youth with BD, studies have reported a significant reduction in both phosphocreatine and inorganic phosphate in the frontal lobe of the brain [19-21]. In contrast to adults, there is evidence that higher manic symptom severity is associated with lower frontal lobe pH levels in youth with BD [20].

The energy within the cell is produced by mitochondria, and this metabolic process generates heat [22]. Without a mechanism to dissipate this heat, brain temperature would rise at a rate of 0.3°C/min [22]. In humans, the primary mechanism for heat dissipation is through inflowing blood, which acts to absorb heat [23]. The human brain receives approximately 20% of total cardiac output relative to comprising 2% of total body mass [24]. Inflowing blood delivers nutrients, energy substrates, and oxygen to the brain tissue, removes metabolic waste, and absorbs heat [24]. Alterations in cerebral blood flow (CBF), which reflects the rate of blood volume supplied to the brain, have been identified in both youth [25, 26] and adults [27, 28] with BD, and there is evidence of mood-related differences in CBF [26, 27]. In addition to heat generation during energy production and metabolism, another major source of heat generation is through the regulation of uncoupling proteins. Uncoupling proteins are located on the inner membrane of the mitochondria and are essential in thermogenesis in response to internal or external temperature changes [29]. A post-mortem study on adults with BD has reported a reduction in uncoupling protein 2 mRNA expression in the prefrontal cortex compared to HC [30]. It provides putative evidence of altered brain thermogenesis in patients with BD.

A recent study on a healthy adult population revealed that brain temperature ranged between 36.1 to 40.9°C in the HC group across different brain regions and pointed out that brain temperature was 0.36°C higher in females in their luteal phases relative to females in their follicular phase and males [31]. A change of 0.3°C to 0.7°C in body temperature was used to clinically monitor the menstrual cycle in women, especially the post-ovulatory luteal phase [32]. In addition, it has been shown that a 0.4°C change in body temperature will trigger a thermoregulatory response to maintain homeostasis [33].

The MRS technique is a validated method of measuring brain temperature non-invasively in humans [34-36]. This method utilizes the linear relationship between the chemical shift of water and temperature while using N-acetyl aspartate (NAA) as a reference due to its temperature-independent chemical shift [34, 37]. To date, the only study that has examined brain temperature in adults with BD used diffusion imaging and observed a non-significant trend of lower brain temperature as compared to controls [38]. However, no other studies have examined this in youth with BD.

In the current proof-of-concept study, we assessed brain temperature in youth with and without BD using MRS within two regions of interest (ROI), the anterior cingulate cortex (ACC) and the precuneus, both of which exhibit altered CBF [28] and brain metabolism in BD [39-41]. In addition, comparing individuals with BD to healthy controls, task-based functional magnetic resonance imaging (MRI) studies utilizing executive and emotional processing tasks have revealed functional alterations in the cingulate cortex and precuneus [42-45]. In a recent study, we reported a potential mismatch between CBF and cerebral rate of oxygen metabolism in BD, suggestive of inefficiency in brain energy homeostasis [46]. Here we examined the association of brain temperature with diagnostic group (BD *vs*. control group (CG)), mood symptom severity, and CBF. We hypothesized that the same factors underlying inefficient brain energy homeostasis would also be implicated in inefficient thermoregulation, resulting in increased brain temperature in youth with BD compared to healthy controls. We further hypothesized that higher brain temperature could be associated with greater mood symptom severity and higher CBF. Secondary analyses examined brain temperature-to-CBF ratio in relation to mood within the BD group.

## 2 MATERIALS AND METHODS

### 2.1 Participants

The current proof-of-concept study adopted the consecutive sampling method for participant recruitment. A total of 62 potential participants were contacted for the study, of which 48 responded and had an informed consent discussion. Of these, 21 did not enroll for the following reasons: Two out of the 48 were not interested in participating and did not indicate a specific reason, two participants had concerns about MRI scans, and six participants expressed initial interest but did not return a signed consent form. Ten participants provided signed informed consent and scheduled a study intake visit but canceled or did not attend. One participant was scheduled but was later determined to have an MRI contraindication. A total of 27 participants attended the intake visit, of which two did not complete the imaging visit due to the onset of the COVID pandemic. The final analyzable sample included 25 participants. All participants are English-speaking youth between the age of 13-20 years old, with 13 in BD and 12 in the CG group. Arterial spin labeling data were missing for one participant, yielding 24 participants for CBF-related analyses. Following a discussion about the study, participants and parents reviewed the informed consent form and had an opportunity to ask questions. Prior to providing written informed consent, all youth participants successfully completed an informed consent assessment, which confirmed their understanding of the study's purpose, risks, and benefits. Written informed consent was obtained from all participants and at least one of their guardian(s) prior to participation. BD participants were recruited from a tertiary subspecialty clinic who meet diagnostic criteria for BD-I (Bipolar I Disorder), BD-II (Bipolar II Disorder), or BD-NOS (Not Otherwise Specified; akin to Other Specified Bipolar and Related Disorders). CG participants were recruited through community advertisements. The CG participants completed the Kiddie Schedule for Affective Disorders and Schizophrenia for School Age Children, Present and Lifetime version (K-SADS-PL) [47], a semi-structured interview, to confirm diagnoses [47]. CG was excluded if participants had lifetime mood or psychiatric disorders, a family history of BD or psychotic disorder (first- and second-degree relatives), or exposure to psychiatric medications in the past three months. Exclusion criteria applied to all participants who were unable to provide informed consent, had contraindications to MRI, neurological or cognitive impairment, or infectious illness within the past 14 days, or had recent alcohol or drug dependence in the past three months. All study procedures were approved by the research ethics board at Sunnybrook Health Sciences Centre (REB 3561/2022). The study was also approved by the Centre for Addiction and Mental Health (CAMH) Research Ethics Board (REB 173/2020), as data analyses were undertaken at CAMH following the relocation of the Centre for Youth Bipolar Disorder.

### 2.2 Psychiatric and Anthropometric Measures

According to the Diagnosis and Statistical Manual of Mental Disorders, fourth edition (DMS-IV) criteria, the K-SADS-PL was employed to ascertain present episodes and lifetime history of psychiatric illness. K-SADS Mania Rating Scale and Depression Rating Scale [48, 49] were used to evaluate diagnosis and symptom severity. BD-NOS was defined based on the COBY(Course and Outcome of Bipolar Illness in Youth) study [50]. All diagnoses were reviewed with a licensed child-adolescent psychiatrist in consensus conferences. The age at which the participants first experienced an episode of mania or hypomania, or were diagnosed with BD-NOS, was defined as the age of BD onset. Race and menstrual cycle were evaluated using self-report. Information on psychotropic medication use and tobacco use was collected during the K-SADS-PL interview. The Family History Screen Interview was completed with participants and/or parents to provide psychiatric history for all first and second-degree relatives [51]. The pubertal stage was assessed using the Pubertal Developmental Scale [52]. All interviews were conducted under the supervision of the senior author.

An anthropomorphic form was used to evaluate the participant’s height (cm), weight (kg), blood pressure, and waist circumference measurements. Height and weight were assessed twice and averaged for precision consideration [53]. Body mass index (BMI) was calculated as kg/m^2^.

### 2.3 Brain Magnetic Resonance Imaging (MRI) Acquisition

All MRI scans took place in a research-dedicated 3 Tesla system (MAGNETOM Prisma, Siemens Healthineers, Erlangen, Germany). High-resolution T1-weighted images were acquired for co-registration purposes using the following parameters: axial three-dimensional magnetization-prepared rapid gradient echo sequence, parallel to the AC-PC line, echo time (TE)/inversion time (TI)/repetition time (TR) = 2.21 ms/900 ms/1800 ms, flip angle = 10, isotropic 1 mm^3^ voxel, 176 sagittal slices, matrix size = 256x256, acquisition time: 4’12”.

Pseudo-continuous arterial spin labeling (PC-ASL) imaging was performed to obtain cortical perfusion levels in absolute units of mL/100 g of tissue /min on a voxel-by-voxel basis and with a spatial resolution of approximately 2.5 x 2.5 x 2.5 mm^3^. The following parameters were applied: post label delay = 1.8 s, TE/TR = 36.76/4100 ms, flip angle = 120°, field of view = 240 mm, 96x96 matrix, 48 slices, and scan duration of 4’27”.

**Fig. (1):**
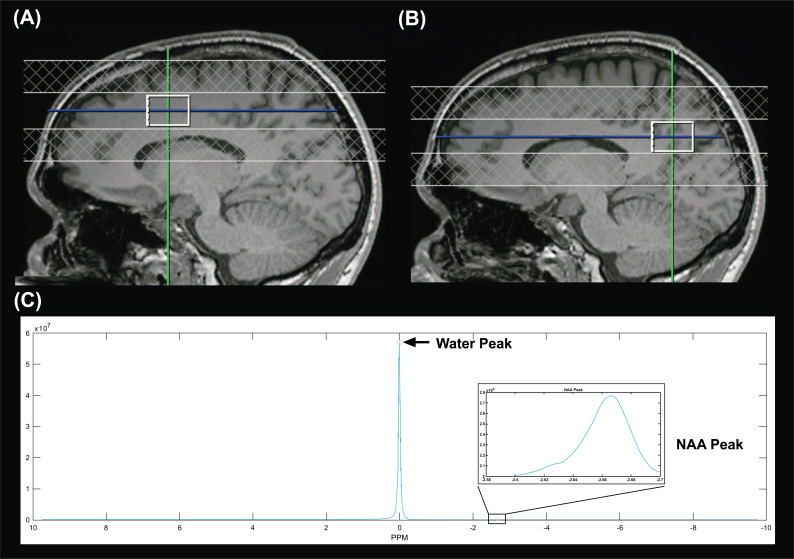
(**A**) The spectroscopic voxel was positioned in the left anterior cingulate and superior frontal, anterior of the precentral gyrus, including medial gray matter and white matter for the ACC. (**B**) The spectroscopic voxel was positioned in the left precuneus, anterior of the parietal-occipital fissure, into the posterior cingulate, including medial gray matter and white matter for the precuneus. (**C**) Spectrum for water peak and NAA peak identification.

Water unsuppressed, single-voxel MR spectroscopy (SVS) data were acquired using a standard clinical Point RESolved Spectroscopy (PRESS) sequence (TE/TR = 30 ms/2200 ms, voxel size of 25 x 18 x 18 mm^3^, 16 averages, 2048 data points). Each ROI was measured eight times for a total acquisition time of 6’10” per ROI. Two ROIs were evaluated: midline anterior cingulum (ACC) and midline precuneus of the left hemisphere. For the ACC (Fig. **1A**), the spectroscopic voxel was positioned in the left anterior cingulate and superior frontal, anterior of the precentral gyrus, including medial gray matter and white matter. For the precuneus (Fig. **1B**), the spectroscopic voxel was positioned in the left precuneus, anterior of the parietal-occipital fissure, into the posterior cingulate, including medial gray matter and white matter. The spectroscopic voxel was positioned carefully to avoid extending out of the brain tissue into the ventricle. The voxel placements for both ROIs were completed by an experienced MRI technologist and confirmed by a senior radiologist.

### 2.4 Brain Imaging Processing

T1-weighted images were processed using the *fsl_anat* script, which included skull-stripping, co-registration to ASL, and a 2mm 152 MNI (Montreal Neurological Institute) standard brain, intensity normalization, and tissue segmentation. ASL data were processed using the command line part of the BASIL toolbox within FSL. The CBF estimates were obtained from the signal difference of consecutive control and blood-labeled images. These estimates were converted to absolute units (mL/100g/min), and spatial smoothing was applied to all CBF maps. ROIs were defined using the Harvard-Oxford atlases available in FSL. To mask the left ACC and precuneus, the corresponding atlas labels in 2mm standard MNI152 space were transformed into ASL space. Specifically, the partial volume corrected CBF maps in native space were used as the reference files for this transformation. Masks in ASL space were then multiplied by a GM mask to restrict the extent of the ROI to GM voxels. CBF values were, in turn, extracted using these masks.

SVS data processing and brain temperature calculations were performed with MATLAB version R2019b employing a locally modified version of the MRS_MRI_libs toolbox and additional custom scripts. Briefly, the pre-processing steps included zero-filling, apodization, and Fourier transformation to obtain the final water-unsuppressed MRS spectrum. Water and NAA peaks in parts per million (ppm) were identified as the local maxima on the spectrum plot (Fig. **1C**). Water-NAA chemical shift differences were used to calculate brain temperature in degrees Celsius (°C) with the following equation: brain temperature (°C) = 310.9-103.2 (Water_ppm_-NAA_ppm_). The calibration coefficients for this calculation were obtained by linearly fitting the results of similar MRS acquisitions in a phantom across a range of temperatures, using a fiber optic temperature sensor for reference. The brain temperature was calculated for each repeated MRS acquisition and averaged to obtain the final estimated brain temperature for each ROI.

### 2.5 Statistical Analyses

All statistical analyses were performed using the SPSS statistic software (IBM; NY, USA), version 27. Shapiro-Wilks test and Levene’s test were employed to check the normality and equal variance assumptions of all continuous variables. Independent t-test and chi-square (χ^2^) tests were performed to investigate group differences for continuous and categorical variables, respectively. The analysis of covariance (ANCOVA) model was employed to evaluate the group differences in brain temperature. A general linear model was used to examine the association between brain temperature and CBF (in the whole sample and each diagnosis group) and mood severity scores within BD. Secondary analysis within the BD group examined the association between brain temperature-to-CBF ratio with the depression rating scale and mania rating scale mood symptom severity score using a general linear model. Associations between CBF and mood symptom severity scores were examined for any significant findings in the secondary analyses to confirm the role of brain temperature in predicting mood. All analyses were controlled for age, sex, and psychiatric medication. Statistical significance was set at α = 0.025 (two-tailed) to account for the 2 temperature ROIs at a critical p-value of 0.05. Sensitivity analysis was carried out to examine group differences in brain temperature among females in the luteal phase, females in the follicular phase, and male groups using analysis of covariance model covarying for age. Effect sizes were reported as Cramér’s V (*V*), Cohen’s d (*d*), or R^2^ and standardized beta (ß) for chi-square, independent t-test, and regression analysis, respectively.

## 3 RESULTS

There were no significant between-group differences in age (BD = 17.2 ± 2.0; CG = 17.6 ± 1.3), race (BD Caucasian = 70%; CG Caucasian = 50%), pubertal status (Tanner stage ≥ 4: BD = 85%, CG = 83%; information missing for 1 participant), or BMI (BD = 23.0 ± 3.5; CG = 20.9 ± 2.6). There was a significantly higher proportion of participants with female sex in the BD group (85%) compared to CG (42%; χ^2^ = 5.0, *p* = 0.03, *V* = 0.5). Clinical characteristics are summarized in Table **1**.

**Fig. (2):**
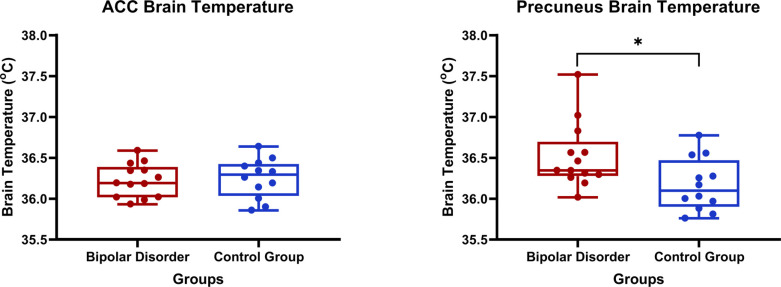
Between-group comparison of brain temperature in the ACC (left) and precuneus (right); For the ACC, no significant group difference was observed (F = 0.92; *p* = 0.35; η_p_^2^ = 0.04). For the precuneus, brain temperature was significantly higher in the BD group compared to CG (F = 10.78; *p* = 0.004; η_p_^2^ = 0.35).

There were no significant between-group differences in ACC temperature (BD: 36.2 ± 0.2°C; CG: 36.2 ± 0.2°C), covarying for age, sex, and psychiatric medication (F = 0.92; *p* = 0.35; η_p_^2^ = 0.04). The temperature in the precuneus was significantly higher in BD (36.5 ± 0.4°C) compared to CG (36.2 ± 0.3°C), covarying for age, sex, and psychiatric medication (F = 10.78; *p* = 0.004; η_p_^2^ = 0.35) (Fig. **2**). This finding remained after correction for multiple comparisons. In addition, the group difference in brain temperature for the precuneus remained significant after removing the outlier from the BD group (F = 5.45; *p* = 0.03; η_p_^2^ = 0.22). Brain temperature was not significantly associated with mood symptom severity or CBF, controlling for age, sex, and medication (Supplementary Tables **S1** and **S2**).

**Fig. (3):**
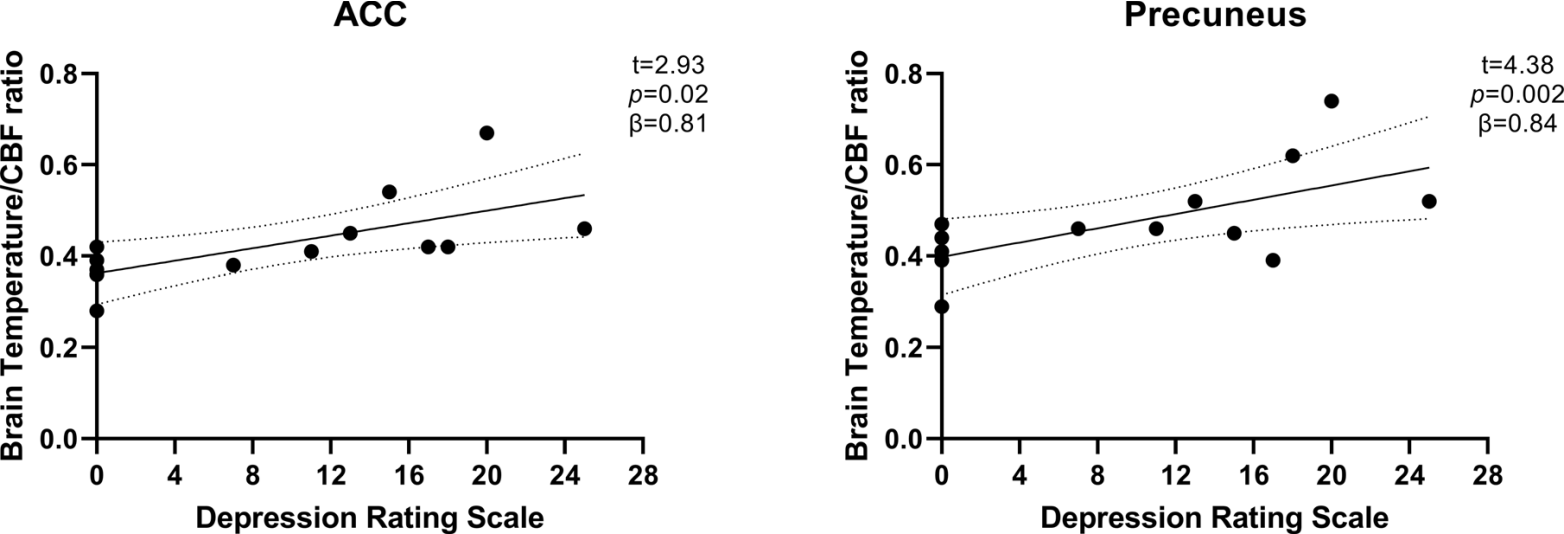
Significant association between temperature-to-CBF ratio and depression rating scales for both the ACC (left) and the precuneus (right). The dotted lines represent the 95 percent confidence interval.

In the secondary analyses within BD, higher brain temperature-to-CBF ratio (ACC: mean = 0.43°C/mL/100g/min ± 0.10; Precuneus: mean = 0.47°C/mL/100g/min ± 0.11) was significantly associated with higher depression symptom severity in both ROIs (Fig. **3**) covarying for age, sex, and psychiatric medication (ACC: *R*^2^ = 0.69, F = 4.51, *p* = 0.03; t = 2.93, *p* = 0.02; ß = 0.81; Precuneus: *R*^2^ = 0.76, F = 6.33, *p* = 0.01; t = 4.38, *p* = 0.002, ß = 0.84). In contrast, there were no significant associations with manic symptom severity (ACC: *R*^2^ = 0.54, F = 2.32, *p* = 0.15; Precuneus: *R*^2^ = 0.28, F = 0.79, *p* = 0.57). A sensitivity analysis related to the temperature-to-CBF ratio findings found non-significant associations of CBF alone with depression symptom severity in the ACC (*R*^2^ = 0.65, F = 3.74, *p* = 0.05) and the precuneus (*R*^2^ = 0.59, F = 2.81, *p* = 0.10) controlling for age, sex, and psychiatric medications. In addition, sensitivity analyses examining brain temperature differences across females in the luteal phase (n = 5), females in the follicular phase (n = 9), and males (n = 9), covarying for age, did not reveal any significant group differences for the ACC (F = 0.88; *p* = 0.43; η_p_^2^ = 0.09) or the precuneus (F = 0.80; p = 0.46; η_p_^2^ = 0.08).

## 4 DISCUSSION

The present study used a novel MRI approach to evaluate brain temperature concerning diagnosis, CBF, and mood symptom severity scores in a youth population with and without BD. We observed significantly higher brain temperature in the BD group compared to the CG in the precuneus. Although associations of brain temperature with CBF and mood severity were not significant, the brain temperature-to-CBF ratio was significantly associated with depression severity in both ROIs (ACC, precuneus). In summary, the results from the current study suggest a reduced thermoregulatory capacity within the BD group that is putatively linked with depression symptom severity. In addition, the current study also supports the utility of the non-invasive MRS technique in assessing brain temperature in BD and potentially in other psychiatric disorders.

Brain temperature is strongly regulated by homeostatic processes related to brain energy metabolism [54]. Oxygen and glucose utilization in the brain is an order of magnitude larger than what would be expected by its mass [55, 56], resulting in intense heat production. Brain temperature is determined by the balance between metabolic heat production and CBF-mediated heat removal [55]. The brain is sensitive to fluctuations in temperature as it has a role in various physiological and neurochemical processes in the brain [57]. The current study revealed significantly higher brain temperature in the precuneus of youth with BD compared to the CG. The precuneus, part of the superior parietal lobe [58], is part of the default-mode network, which is known to have altered function in BD [59]. Prior literature has observed altered cerebral metabolism and CBF in the precuneus among patients with psychiatric disorders. Two nuclear medicine studies have revealed reduced glucose metabolism in the precuneus [60] and reduced CBF in the parietal lobe in depressed adults with BD compared to healthy controls [61]. Studies on adults with major depressive disorder have found reduced CBF in the precuneus [62] and an association between a greater number of depressive episodes with significantly lower CBF in the precuneus [63]. Dysregulation of both brain energy metabolism and CBF might alter the balance between heat production and removal, ultimately affecting brain thermoregulation [64, 65]. The current study did not reveal any significant association between brain temperature and CBF metrics in BD. In a healthy brain, the temperature depends on the balance between heat production and CBF-mediated heat removal. One can speculate that the lack of association might be due to differences in metabolism in BD, specifically increased perfusion demands and, in turn, a compensatory failure to meet them. Essentially, metabolic demands may outpace the ability of CBF to dissipate heat.

Although brain temperature, CBF, and mood symptom severity scores were not associated with one another, the current study found that a higher brain temperature-to-CBF ratio in the BD group was associated with higher depression symptom severity scores in both ROIs. As brain temperature is reflective of cerebral energy metabolism, a higher ratio might indicate reduced thermoregulation as a result of an increase in metabolic heat production, reduced CBF, or the combination. The ACC is essential in emotion regulation and cognitive control, both of which are impaired among individuals with BD [66]. Prior studies using MRS and nuclear medicine imaging reported alteration in brain energy metabolism [60, 67-69] and reduced CBF in the ACC in depressed adults with BD compared to healthy controls [28]. For the precuneus, lower metabolism was associated with higher depression severity in adults with the major depressive disorder [70-72].

The present findings are constrained by a number of limitations. First, the cross-sectional design precludes inferences regarding temporal associations among observed findings. Second, although we controlled psychiatric medication use, we could not completely eliminate the possibility of residual confounding effects. Third, the sample size is limited; for this reason, we cannot exclude the possibility of additional findings of smaller effect sizes than those in the current study and cannot examine additional covariates or explore the effect of individual psychiatric medications. Fourth, the current study focused solely on NAA as the reference peak. NAA is comparatively abundant relative to creatine and choline (especially in water-unsuppressed acquisitions), can be more reliably localized than other metabolites, and is the most commonly used metabolite for thermometry [34, 37, 73, 74].

Nonetheless, adding additional metabolic peaks, such as creatine and choline, would be potentially informative in future studies with larger samples. In addition, the MRS acquisition was performed on the ACC, followed by the precuneus. Therefore, it is theoretically possible that the MRI radio frequency increased brain temperature. MRS uses relatively low radio frequency energy, particularly our water-unsuppressed acquisitions that forgo the usual water suppression pulses. Although we do not expect the MRI radio frequency energy deposition to have an appreciable effect on the brain temperature measurement, we cannot completelyeliminate this possibility, given the lack of random ordering in our design. Lastly, because the study examined water-unsuppressed MRS data, we could not examine specific molecules, such as lactate, that are relevant to energy metabolism.

## CONCLUSION

To conclude, the current study provides putative evidence of reduced thermoregulation in youth with BD compared to CG, with relevance to depression symptom severity in the BD group. Such findings suggest the potential benefit of examining brain temperature alongside CBF, as this may provide unique insights that are not possible when only evaluated individually. In addition, the results of the current study pointed out the value of using the non-invasive MRS technique for assessing brain temperature in BD and potentially in other psychiatric disorders. The fact that the ratio of brain temperature to CBF is associated with symptom severity in youth with BD suggests that future experimental medicine trials could potentially engage this objective treatment target. Indeed, the current finding is well aligned with contemporary conceptualizations of BD as an illness of impaired energy metabolism and vascular dysfunction. Overall, with its limitations acknowledged, the current study adds to the sparse literature on brain thermoregulation in BD. Future prospective studies with larger samples are needed to confirm the current findings, incorporating brain energy metabolites and examining temporal associations of present findings.

## Figures and Tables

**Table 1:** Clinical characteristics.

-	**BD (n = 13)**
** Psychiatric Characteristics**	
BD-I	7 (54%)
BD-II	3 (23%)
BD-NOS	3 (23%)
Age of BD Onset	15.5 ± 1.8
Mania Rating Scale	5.2 ± 6.3
Depression Rating Scale	9.7 ± 9.0
** Lifetime Clinical Characteristics**	
Lifetime Psychosis	6 (46%)
Lifetime Suicide Attempts	5 (38%)
Lifetime Self-injurious Behaviour	7 (54%)
Lifetime Suicidal Ideation	8 (62%)
Lifetime Physical and/or Sexual Abuse	1 (8%)
Lifetime Psychiatric Hospitalization	7 (54%)
** Lifetime Comorbid Diagnoses**	
ADHD	3 (23%)
Anxiety Disorder	11 (85%)
Number of Anxiety Disorders	2.5 ± 2.0
Conduct Disorder	0 (0%)
Oppositional Defiant Disorder	3 (23%)
Substance Use Disorder	3 (23%)
Nicotine Use	5 (38%)
** Current Medications**	
Second Generation Antipsychotics	8 (62%)
Lithium	2 (15%)
Non-SSRI Antidepressants	0 (0%)
SSRI Antidepressants	0 (0%)
Stimulants	1 (8%)
Any Medication	9 (70%)
** Family Psychiatric History**	
Mania/Hypomania	5 (38%)
Depression	10 (77%)
Anxiety	9 (70%)
ADHD	4 (31%)

**Note**: Results are reported as mean ± standard deviation (SD) or percentage (%) unless otherwise specified. BD = Bipolar disorder; NOS = Not otherwise specified; ADHD = Attention deficit-hyperactivity disorder; SSRI = Selective serotonin reuptake inhibitor.

-Missing cases (n): Lifetime Suicide Attempts (1); Lifetime Self-injurious Behaviour (1); Lifetime Suicidal Ideation (1); Lifetime Physical and/or Sexual Abuse (2); Legal History (1).

-For the control group (%): Legal History (8%); Lifetime Anxiety Disorder (25%); Family Psychiatric History of Depression (33%); Family Psychiatric History of Anxiety (8%).

## Data Availability

The datasets used and/or analyzed during the current study are available from the corresponding author on reasonable request. The data are not publicly available due to privacy or ethical restrictions.

## LIST OF ABBREVIATIONS

ACC: Anterior Cingulate Cortex
BD: Bipolar Disorder
CBF: Cerebral Blood Flow
CG: Control Group
MRI: Magnetic Resonance Imaging
MRS: Magnetic Resonance Spectroscopy
ROI: Regions of Interest
