# Supplementary material for: Measuring Brain Temperature in Youth Bipolar Disorder Using a Novel Magnetic Resonance Imaging Approach: A Proof-of-concept Study
Source: Curr Neuropharmacol. 2023 May 12;21(6):1355–66. doi: 10.2174/1570159X21666230322090754 (PMC10324328; doi:10.2174/1570159X21666230322090754)
Supplement: Supplementary file 1 [file CN-21-1355_SD1.pdf]

Supplementary Material

Measuring Brain Temperature in Youth Bipolar Disorder Using a Novel Magnetic Resonance Imaging Approach: A Proof-of-concept Study

Yi Zou[1,2], Chinthaka Heyn[3,4], Anahit Grigorian[2], Fred Tam[4], Ana Cristina Andreazza[1,5], Simon J. Graham[4,6], Bradley J. Maclntosh[6,7,8] and Benjamin I. Goldstein[1,2,5,*]

[1]Department of Pharmacology, University of Toronto, Toronto, ON, Canada; [2]Centre for Youth Bipolar Disorder, Centre for Addiction and Mental Health, Toronto, ON, Canada; [3]Department of Medical Imaging, Sunnybrook Health Sciences Centre, Toronto, ON, Canada; [4]Physical Sciences Platform, Sunnybrook Research Institute, Toronto, Canada; [5]Department of Psychiatry, University of Toronto, Toronto, M5T 1R8, ON, Canada; [6]Department of Medical Biophysics, University of Toronto, Toronto, ON, Canada; [7]Heart and Stroke Foundation, Canadian Partnership for Stroke Recovery, Sunnybrook Research Institute, Toronto, ON, Canada; [8]Hurvitz Brain Sciences Program, Sunnybrook Research Institute, Toronto, ON, Canada

Supplementary Table 1. Association between brain temperature with mood symptom severity scores within BD.

	**ACC**	**Precuneus**
**MRS**	R^2=0.15, F=0.35, *p*=0.84, ß=-0.18	R^2=0.40, F=1.34 , *p*=0.33, ß=-0.19
**DRS**	R^2=0.20, F=0.51, *p*=0.73, ß=-0.40	R^2=0.39, F=1.29, *p*=0.35, ß=-0.19

Note: Brain regions are restricted to the left-brain hemisphere. ACC= anterior cingulate cortex; MRS=Mania rating score; DRS=Depression rating score. Standardized ß value was reported for the mood scores.

Supplementary Table 2. Association between brain temperature with CBF.

	**ACC**	**Precuneus**
**Whole Sample**	R^2=0.10, F=0.50, *p*=0.73, ß=-0.01	R^2=0.12, F=0.65, *p*=0.63, ß=0.05
**CG**	R^2=0.22, F=0.66, *p*=0.60, ß=-0.25	R^2=0.38, F=1.40, *p*=0.32, ß=0.09
**BD**	R^2=0.32, F=0.93, *p*=0.50, ß=0.53	R^2=0.40, F=1.36, *p*=0.33, ß=0.18

Note: Brain regions are restricted to the left-brain hemisphere. CBF=Cerebral blood flow; CG= Control group; BD=Bipolar disorder. Standardized ß value was reported for the CBF dependent variable.

1875-6190/23 $65.00+.00 **© 2023 Bentham Science Publishers**
